# Cardiovascular magnetic resonance for the assessment of patients undergoing transcatheter aortic valve implantation: a pilot study

**DOI:** 10.1186/1532-429X-13-82

**Published:** 2011-12-27

**Authors:** Alessio La Manna, Alessandra Sanfilippo, Davide Capodanno, Antonella Salemi, Gesualdo Polizzi, Wanda Deste, Glauco Cincotta, Alessandra Cadoni, Anna Marchese, Michele Figuera, Gian P Ussia, Rosetta Pittalà, Carmelo Privitera, Corrado Tamburino

**Affiliations:** 1 Division of Cardiology, Ferrarotto Hospital, (via Citelli 1), Catania, (95100), Italy; 2 Excellence Through Newest Advances (ETNA) Foundation, (Viale XX Settembre 70), Catania, (95129), Italy; 3 Radiology Unit, Vittorio Emanuele Hospital, (via Plebiscito 628), Catania, (95122), Italy

**Keywords:** cardiovascular magnetic resonance, transcatheter aortic valve implantation, trans-thoracic echocardiography

## Abstract

**Background:**

Before trans-catheter aortic valve implantation (TAVI), assessment of cardiac function and accurate measurement of the aortic root are key to determine the correct size and type of the prosthesis. The aim of this study was to compare cardiovascular magnetic resonance (CMR) and trans-thoracic echocardiography (TTE) for the assessment of aortic valve measurements and left ventricular function in high-risk elderly patients submitted to TAVI.

**Methods:**

Consecutive patients with severe aortic stenosis and contraindications for surgical aortic valve replacement were screened from April 2009 to January 2011 and imaged with TTE and CMR.

**Results:**

Patients who underwent both TTE and CMR (n = 49) had a mean age of 80.8 ± 4.8 years and a mean logistic EuroSCORE of 14.9 ± 9.3%. There was a good correlation between TTE and CMR in terms of annulus size (R^2 ^= 0.48, p < 0.001), left ventricular outflow tract (LVOT) diameter (R^2 ^= 0.62, p < 0.001) and left ventricular ejection fraction (LVEF) (R^2 ^= 0.47, p < 0.001) and a moderate correlation in terms of aortic valve area (AVA) (R^2 ^= 0.24, p < 0.001). CMR generally tended to report larger values than TTE for all measurements. The Bland-Altman test indicated that the 95% limits of agreement between TTE and CMR ranged from -5.6 mm to + 1.0 mm for annulus size, from -0.45 mm to + 0.25 mm for LVOT, from -0.45 mm^2 ^to + 0.25 mm^2 ^for AVA and from -29.2% to 13.2% for LVEF.

**Conclusions:**

In elderly patients candidates to TAVI, CMR represents a viable complement to transthoracic echocardiography.

## Background

Aortic stenosis is one of the most common heart diseases worldwide, especially in the elderly [1]. In recent years, transcatheter aortic valve implantation (TAVI) has emerged as a valuable alternative to surgical aortic valve replacement in patients at high surgical risk because of age and/or comorbidities [2,3]. Before TAVI, global assessment of cardiac function and accurate measurement of the aortic root are key to determine the correct size and type of the prosthesis [4].

Trans-thoracic echocardiography (TTE) is the recommended method for the preliminary assessment of aortic stenosis and left ventricular function. TTE provides an appraisal of aortic root sizes, stenosis severity and left ventricular function [5,6]. However, TTE is limited by poor acoustic window and inter-observer variability. The use of trans-esophageal echocardiography (TEE) allows a more precise measurement but, being a semi-invasive technique [7], it is often poorly tolerated by elderly patients. In addition, accurate definition of the valve planimetry with TEE is limited when the aortic leaflets are heavily calcified. Therefore, in the screening phase for assessing the eligibility to TAVI, increasing emphasis has been given to the use of non-invasive, real time, three-dimensional imaging techniques such as 3-dimensional echocardiography, multidetector row computer tomography (MDCT) and cardiovascular magnetic resonance (CMR) [8,9]. Many validation studies of MDCT versus echocardiography have been published [10-13]. Importantly, while the high spatial resolution of MDCT enables accurate quantification of the aortic valve area, the use of iodinated contrast media may limit its application in the elderly population.

Recently, CMR has been increasingly used for the assessment of aortic valve area in patients with aortic stenosis [14-16]. CMR is a non invasive, radiation-free imaging modality which allows not only a detailed visualization of cardiac structures, but also functional assessment including wall motion analysis, quantification of cardiac function and myocardial tissue characterization. The steady-state free precession (SSFP) techniques allow direct visualization of the aortic valve area for planimetry in any chosen plane with excellent image quality. To date, however, no exhaustive data on the use of CMR prior to TAVI have been published. The aim of this study was to compare CMR and TTE for the assessment of aortic valve measurements and left ventricular function in high-risk elderly patients submitted to TAVI.

## Methods

### Study Population and Design

A total of 103 consecutive patients with severe aortic stenosis and high risk and/or contraindications for surgical aortic valve replacement were screened from April 2009 to January 2011. Clinical outcomes of a proportion of patients undergoing TAVI have been reported elsewhere [17]. Exclusion criteria for this study were general contraindications to CMR [18]. The institutional ethics committee approved the study protocol and all patients gave informed consent.

Patients were imaged with TTE and CMR (within 3 to 5 days from TTE), whenever feasible. The following measurements were obtained with each imaging technique: 1) aortic root sizes (annulus, sinus, sinotubular junction, ascending aorta); 2) aortic valve area; 3) aortic peak velocity; 4) left ventricular morphology and function.

### Two-dimensional trans-thoracic echocardiography

Patients underwent preoperative TTE with an Acuson Sequoia (Siemens C 512 System Images). All examinations were stored on CD and analyzed by the same experienced echocardiographer. Aortic annulus, sinus of Valsalva and sinotubular junction were sized using bi-dimensional measurement during the diastole in the long parasternal axis. Measurements were performed between the insertions of the right and non-coronary leaflets to the aortic annulus and the mean value from three consecutive measurements was used. The ascending aorta diameter was measured in the same projection at a distance of 35 mm from the aortic annulus; the measurement was performed from the beginning of the anterior wall up to the end of the posterior wall of the aorta.

**Figure 1:**
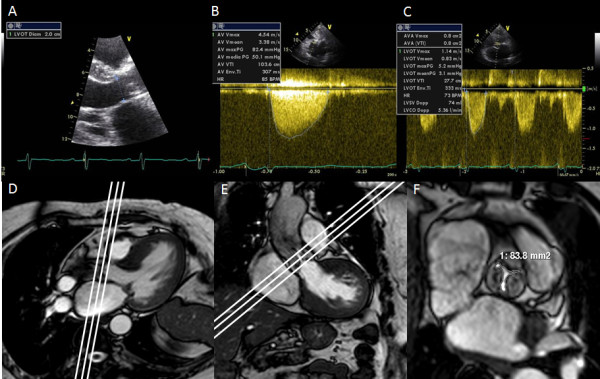
**Aortic valve area measurement by trans-thoracic echocardiography (TTE) and cardiovascular magnetic resonance (CMR)**. Aortic valve area measured by continuity equation TTE approach: Left ventricular outflow tract (LVOT) diameter (**A**); transaortic peak velocity (**B**); aortic velocity-time integral (**C**). Oblique sagittal view of the aortic outflow tract, with the CMR slice position indicated by three white lines orthogonal to the stenotic jet (**D**). Oblique transaxial view of the aortic outflow tract, with CMR slice position indicated by three white lines (**E**). Cross-sectional view of a severely stenotic aortic valve; the white line denotes the aortic valve area (**F**).

The left ventricular outflow tract (LVOT) diameter was measured in mid-systole in the para-sternal long-axis view (Figure 1A) proximally to the position of the pulse wave Doppler data (5 mm below above valve), then converted to LVOT area (A_LVOT_) according to the following equation: A = Π r^2^. Peak velocities and velocity time integral (Figure 1B and 1C), calculated with the use of the resident software at the time of imaging, were used to calculate pressure gradients according to the modified Bernoulli equation (ΔP = 4V^2^) and aortic valve area (AVA) was obtained according to the continuity equation approach [AVA = A_LVOT _(VTI_LVOT_/VTIvalve)]. End-diastolic and end-systolic left ventricular volumes were determined using the biplane Simpson's method according to the recommendation of the American Society of Echocardiography [5]. Left ventricular ejection fraction (LVEF) was calculated as (end-diastolic volume - end-systolic volume)/end-diastolic volume. All calculations were blinded to the CMR data.

### Cardiovascular Magnetic Resonance

All CMR studies were performed with a Philips Achieva 1.5 Tesla Magnetic Resonance Imaging scanner with a 5-element, phased-array coil (Philips Healthcare, Best, The Netherlands). To assess ventricular function, cine images of the entire left ventricle were acquired using an ECG-gated balanced SSFP pulse sequence. Following this step, an intravenous bolus dose of 0.2 mmol/kg body weight of gadobutrol (Gadovist, Bayer Schering Pharma, Berlin, Germany) was administered at a rate of 3 ml/s by a power injector (Medrad Spectris Solaris, Medrad, USA). Ten minutes after gadolinium injection, a 'Look Locker' sequence was performed to obtain the most appropriate inversion time (TI) to null the signal intensity of normal myocardium. Late gadolinium enhancement (LGE) images were then acquired using the following images parameters: fast gradient echo, repetition time (TR) 6.1 ms, echo time (TE) 3 ms, flip angle (FA) 25°, field of view (FOV) 320 mm, slices thickness 10 mm, acquired in the left ventricular (LV) short axis over 2 RR intervals and no interslice gap. The CMR images were analysed off-line using a commercial software (Philips Medical Systems Extended MK Word Space Version 2.6.3.1) by an experienced observer blinded to echocardiographic and clinical data. For assessment of left ventricular function, the end-diastolic and end-systolic cine frames were identified for each slice and the endocardial and epicardial borders were manually traced. The end-diastolic and end-systolic volumes were then calculated using the Simpson's rule (i.e. sum of cavity sizes across all continuous slices). As per echocardiography, LVEF was calculated as (end-diastolic volume - end-systolic volume)/end-diastolic volume. The LGE was evaluated on the basis of visual assessment in short-axis slices. LGE was further characterized by spatial location, pattern, and LGE quantification (1-25%, 25-50%, 50-75%, > 75%) [19].

For aortic valve visualisation and measurement, ECG gating and parallel imaging were used, with SSFP cine images acquired during expiratory breath-holds. Cine series of the LV outflow tract in two orthogonal planes (oblique transverse and oblique coronal: Figures 1D and 1E) were obtained in all patients to visualize the systolic jet originating from the stenotic valve. Imaging planes for planimetry were chosen perpendicular to the stenotic jet, as deduced from the area of signal loss due to turbulent flow at valve orifice level. Serial short-axis cines were acquired (5-mm slice thickness) with no gap until the entire aortic root was imaged. In the cross-sectional planes, planimetry of the AVA was performed in all systolic images to determine the maximum AVA and amplitude (Figure 1F). Using the same planes on the aortic valve, images were acquired with through-plane velocity encoding. The imaging parameters were as follows: TR 5 ms, TE 3 ms, reconstruction matrix 256, FA 12°, recon voxel size 1.25 mm^2^; slice thickness = 8 mm; velocity encoding value (venc) ≥ 150 cm/s (with concomitant gradient correction). In the sagittal and coronal LVOT planes, end diastolic measurements were made at 3 levels: 1) the level of the aortic annulus; 2) the level of the maximum diameter across the sinuses; 3) at the sinotubular junction and 4) at the ascending aorta. LVOT was measured in mid-systole at 1 cm to the aortic root.

### Statistical Analysis

Quantitative data were presented as mean ± standard deviations and were compared using the Student t test. Categorical data were presented as counts and percentages. The correlation between CMR and TTE was analysed by simple linear regression with 95% confidence intervals (CI). Agreement was established by the Bland-Altman test. A two-sided p value of < 0.05 was considered to indicate statistical significance. All data were processed using the Statistical Package for Social Sciences, version 15 (SPSS, Chicago, IL, USA).

## Results

**Figure 2:**
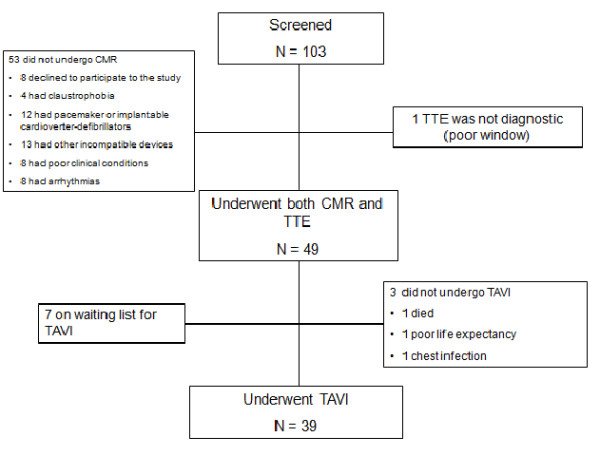
**Study flow chart**. Reasons for excluding patients from cardiovascular magnetic resonance or trans-thoracic echocardiography.

Of 103 patients screened, 53 did not undergo CMR for the reasons listed in Figure 2. One patient did not undergo TTE due to a poor acoustic window and was further excluded from this analysis. Patients who underwent both TTE and CMR (n = 49) had a mean age of 80.8 ± 4.8 years and a mean logistic EuroSCORE of 14.9 ± 9.3%. All patients were in NYHA class III-IV and 33% of them were previously hospitalized for congestive heart failure (Table 1). All patients well tolerated both the TTE and CMR examinations. The images quality was diagnostic in 100% of exams. Gadolinium was not used in three patients due to severe renal failure (creatinine clearance < 30 ml/min). Evidence of LGE was present in 17 (37%) of 46 cases. Causes for LGE were ischemic in 14 cases, non-ischemic in 2 cases and mixed in 1 case. Three patients with an ischemic LGE pattern had no history of previous myocardial infarction. No clinical adverse event was recorded during performance of TTE or CMR.

**Table 1:** Patients Characteristics

Patients	49
Male, n (%)	21 (42.9)
Age, (mean ± DS)	80.8 ± 4.8
Log EuroScore, (mean ± DS)	14.9 ± 9.3
BMI, (mean ± DS)	27.6 ± 5.2
*Symptoms*	
Syncope, n (%)	11 (22.4)
Unstable Angina, n (%)	16 (32.7)
Hospitalization for heart failure, n (%)	16 (32.7)
Dyspnoea, n (%)	42 (85.7)
*Risk Factors*	
Diabetes, n (%)	7 (14.3)
Hypercholesterolemia, n (%)	21 (42.9)
Smoker, n (%)	7 (14.3)
Ex smoker, n (%)	6 (12.2)
History of CAD, n (%)	11 (22.4)
Cirrhosis, n (%)	1 (2)
Renal failure (creatinine > 2 mg/dL), n (%)	6 (12.2)
COPD, n (%)	15 (30.6)
Chronic obstructive arterial disease, n (%)	2 (4.1)
Previous CABG, n (%)	3 (6.1)
Previous PCI, n (%)	19 (38.7)
Previous MI, n (%)	10 (20.4)
Previous TIA, n (%)	6 (12.2)
Previous Stroke, n (%)	4 (8.2)

BMI: body mass index; CAD: coronary artery disease; COPD: chronic obstructive pulmonary disease; CABG: coronary artery by-pass graft; PCI: percutaneous coronary intervention; MI: myocardial infarction; TIA: transient ischemic attack.

**Figure 3:**
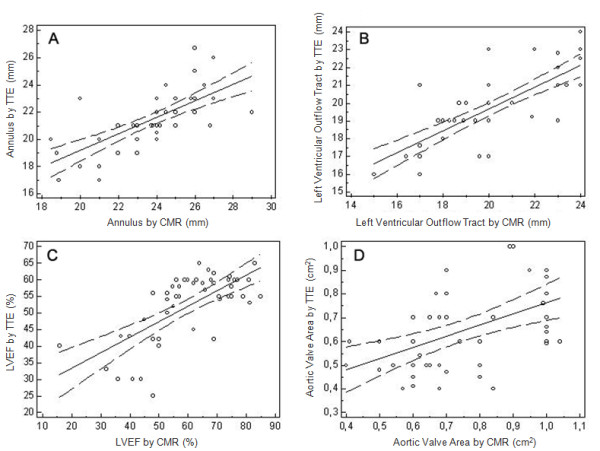
**Scattered plot of trans-thoracic echocardiography (TTE) and cardiovascular magnetic resonance (CMR)**. Correlation analysis of aortic annulus (**A**), left ventricular outflow tract (LVOT) (**B**), left ventricular ejection fraction (LVEF) (**C**) and aortic valve area (AVA) (**D**) measured by TTE and CMR.

The mean velocity aortic peak and regurgitation fraction by CMR were 3.9 **± **0.8 m/s and 11.7 ± 8.9%, respectively. Trans-aortic maximum and mean gradient evaluated by TTE were 92.1 ± 24.5 mmHg and 55.9 ± 16.2 mmHg with one (2%) case of severe aortic regurgitation and 8 (16%) cases of moderate regurgitation. There was a good correlation between TTE and CMR in terms of annulus size (y = 7.15 + 0.60x, R^2 ^= 0.48, p < 0.001, Figure 3A), LVOT (y = 7.37 + 0.61x, R^2 ^= 0.62, p < 0.001, Figure 3B) and LVEF (y = 23.98 + 0.47x, R^2 ^= 0.47, p < 0.001, Figure 3C) and a moderate correlation in terms of AVA (y = 0.29 + 0.47x, R^2 ^= 0.24, p < 0.001, Figure 3D) and peak velocity (y = 3.00 + 0.44x, R^2 ^= 0.28, p < 0.001) (Table 2).

**Table 2:** Correlation between TTE and CMR

	Regression Equation	R^2^	P
EDV (ml)	y = 10.8264 + 0.6053 x	0.50	< 0.001
ESV (ml)	y = 16.0854 + 0.5269 x	0.57	< 0.001
EF (%)	y = 23.9774 + 0.4687 x	0.47	< 0.001
LVOT (mm)	y = 7.3743 + 0.6149 x	0.62	< 0.001
Ascending aorta (mm)	y = 4.4615 + 0.9059 x	0.75	< 0.001
Annulus (mm)	y = 7.1490 + 0.6025 x	0.48	< 0.001
Sinus of Valsalva (mm)	y = 7.0782 + 0.7619 x	0.58	< 0.001
Sinotubular junction (mm)	y = 4.1418 + 0.8444 x	0.59	< 0.001
AVA (cm^2^)	y = 0.2931 + 0.4723 x	0.24	< 0.001
Peak velocity (m/s)	*y = *2,9956 + 0,4350 x	0.28	< 0.001

TTE: trans-thoracic echocardiography; CMR: cardiovascular magnetic resonance; EDV: end diastolic volume; ESV: end-systolic volume EF: ejection fraction; LVOT: left ventricular outflow tract; AVA: aortic valve area.

**Figure 4:**
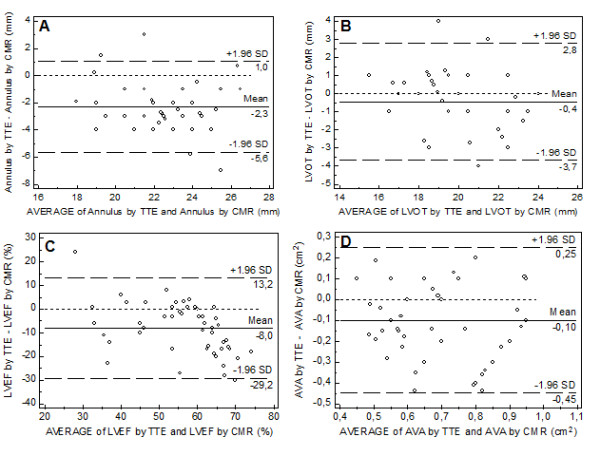
**Agreement of trans-thoracic echocardiography (TTE) and cardiovascular magnetic resonance (CMR) by Bland-Altman analysis**. Bland-Altman analysis for aortic annulus (**A**), left ventricular outflow tract (LVOT) (**B**), left ventricular ejection fraction (LVEF) (**C**) and aortic valve area (AVA) (**D**) measured by TTE and CMR.

However, CMR generally tended to report larger values than TTE for all measurements, including annulus size (absolute difference + 2.3 mm, relative difference + 11%), LVOT (absolute + 0.5 mm, relative difference + 3%), LVEF (absolute difference + 8%, relative difference + 15%), AVA (absolute difference 0.10 mm^2^, relative difference 16%) and peak velocity (absolute difference 0.8 m/s, relative difference + 21%) (Table 3). The Bland-Altman test indicated that the 95% limits of agreement between TTE and CMR ranged from -5.6 mm to + 1.0 mm when assessing annulus size (Figure 4A), from -0.45 mm to + 0.25 mm when assessing LVOT (Figure 4B), from -0.45 mm^2 ^to + 0.25 mm^2 ^when assessing AVA (Figure 4C), from -29.2% to 13.2% when assessing LVEF (Figure 4D) and from -0.64 m/s to + 2.18 m/s when assessing peak velocity.

**Table 3:** Comparison between TTE and CMR measurements

	TTE	CMR	P
EDV (ml)	103.6 ± 41.4	153.3 ± 48	< 0.001
ESV (ml)	50.3 ± 29.2	64.9 ± 42	< 0.001
EF (%)	52.1 ± 10.1	60.1 ± 14.8	< 0.001
LVOT (mm)	19.8 ± 2.08	20.3 ± 2.6	< 0.001
Ascending aorta (mm)	35.8 ± 4.1	34.5 ± 3.9	< 0.001
Annulus (mm)	21.4 ± 1.9	23.7 ± 2.2	< 0.001
Sinus of Valsalva (mm)	30.5 ± 3.3	30.7 ± 3.3	< 0.001
Sinotubular junction (mm)	26.3 ± 3.3	26.3 ± 3.0	< 0.001
AVA (cm^2^)	0.64 ± 0.17	0.74 ± 0.18	< 0.001
Peak velocity (m/s)	3.9 ± 0.8	4.7 ± 0.6	< 0.001

TTE: trans thoracic echocardiography; CMR: cardiovascular magnetic resonance; EDV: end diastolic volume; ESV: end-systolic volume; EF: ejection fraction; LVOT: left ventricular outflow tract; AVA: aortic valve area.

## Discussion

This study adds to the current understanding on imaging appraisal of aortic stenosis with the following observations: 1) in elderly patients, CMR is feasible in the majority of cases, despite potential limitations attributable to age and comorbidities. Common reasons for not undergoing CMR were presence of incompatible devices, claustrophobia, poor clinical conditions and severe arrhythmias; 2) when feasible, CMR provided diagnostic information and adequate sizing in all patients; conversely, LGE could not be assessed in 6.1%; 3) as compared to echocardiography, CMR tends to provide numerically similar measurements in terms of annulus size, LVOT, and AVA; conversely, CMR generally detects larger values of LVEF.

In this study, we comprehensively assessed the correlation and agreement of two different imaging methods in evaluating elderly patients with severe aortic stenosis referred to TAVI. Importantly, general contraindications to CMR did not allow the performance of the test in all screened patients. These inherent limitations, along with attended costs, suggest that CMR should be used only in selected cases, especially in case of questionable results from first-level investigations. Patients submitted to CMR successfully completed the imaging acquisition protocol in all cases, despite an average age of 80 years. In contrast, TTE was not diagnostic in only one patient due to a poor acoustic window.

Echocardiography is the reference standard for the initial preoperative evaluation of aortic stenosis. Several parameters need to be considered in the preoperative assessment of aortic stenosis patients, including stenosis degree, various aortic measurements and left ventricular function [5]. Regarding the aortic gradient assessment, potential limitations of TTE may result from misalignment of jet and ultrasound beam, recording of mitral regurgitation jet, neglect of an elevated proximal speed, or pressure recovery phenomenon [5]. In addition, in patients with technically limited images from TTE or TEE, current guidelines recommend characterization of native and prosthetic cardiac valves, including planimetry of stenotic disease and quantification of regurgitant disease by CMR [20]. Reant et al. showed that CMR planimetry of the AVA is a noninvasive and reproducible technique to evaluate stenotic aortic valves which can be used as an alternative to echocardiography or cardiac catheterisation [21]. Similarly, Pouler et al. proved that both planimetry and continuity equation-based measurements of AVA by CMR are equally accurate [22]. However, similar to TEE, CMR-derived AVA is larger by planimetry than by continuity equation. This is consistent with the notion that the anatomical maximum opening of a stenotic aortic valve is larger than the size of the functional vein contract [22]. Consistently with these assumptions, we observed a good correlation between TTE and CMR, with an absolute mean difference of 0.10 mm^2 ^and only a slight tendency to detect larger areas with CMR.

In patients who are candidate to TAVI, much emphasis is given to aortic annulus sizing, as it implies the choice of type and size of the valve prosthesis. Measurements are usually performed with TTE or TEE, but comparisons between these methods are rare and controversial [23,24]. Recently, it has been suggested that MSCT could also provide detailed information on the shape and length of the aortic annulus [10], but comparisons between MSCT and echocardiographic measurements are not exhaustive. Messika-Zeitoun et al. [25] reported that in TAVI patients, measurements of the aortic annulus using TTE, TEE and MSCT were close but not identical, and the method used has important potential clinical implications on the TAVI strategy. Due to the lack of a gold standard and waiting for further evidence, a strategy based on TEE measurements may provide good clinical results. In this context, CMR may represent a valuable alternative to both TTE and MSCT, with the advantages of clear visualization of the aorta with freedom from ionizing radiation and iodinated contrast-induced effects. Comparative data between CMR and different imaging techniques in evaluating patients referred to TAVI are sparse. Koos et al. [26] recently compared annulus measurements by MSCT, TEE and non-contrast-enhanced CMR using navigator-gated 3-D whole-heart acquisition, showing a good correlation between MSCT and CMR measurements, while the annulus diameters assessed by TEE were significantly smaller than coronal aortic annulus diameters assessed by CMR. This is consistent with our study, which used a different CMR protocol, showing that annulus size adequately correlates with TTE, with an absolute mean difference of + 2.3 mm. In our study, patients did not undergo TEE and MSCT and a formal comparison in terms of feasibility, correlation and agreement with these techniques is not possible at this time. However, considering the age of our study patients and the fact that they have been submitted to a diagnostic examination (CMR) being added to TTE, we felt that the implementation of further diagnostic second-level tests (TEE, MSCT) could have limited the compliance of our enrolled participants. In addition in our centre, as others centres, the TEE is not used routinely for pre- and intra-operative evaluation.

CMR is the gold standard for the calculation of ventricular volumes and LVEF, being recommended especially in cases of heart failure, myocardial infarction, cardiomyopathy, and particularly in presence of a poor acoustic window or discrepancies between different methodologies [20]. In fact, the accuracy of left ventricular volumes and LVEF with two-dimensional echocardiography is limited by image position, geometric assumption, and boundary tracing errors [27]. In our study, LVEF and volumes by TTE were significantly lower than those calculated by CMR. This is probably due to the limitations listed above that are likely to be amplified in this patient population (e.g. elderly with chronic obstructive pulmonary disease, heavy calcifications and/or unfavorable chest conformation). These caveats could be addressed by three-dimensional echocardiography, as noted by Jenkins et al. [28]. However, even three dimensional TTE presents some limitations. Shimada et al. [29] performed a meta-analysis of studies comparing left ventricular volumes and or LVEF between three-dimensional TTE and CMR, showing that three-dimensional TTE systematically underestimates these measures in subgroups such as women and patients with co-existing cardiac disease, whereas use of semiautomatic tracking and matrix-array transducers is needed to counteract this underestimation. Given the above limitations, the usefulness of CMR as an option to reliably estimate volumes and LVEF in patients referred to TAVI (e.g. those with low-flow low-gradient aortic stenosis) is plausible and deserves further investigation.

Finally, CMR allows a precise definition of the infarcted areas, if any, and accurate detection of fibrosis secondary to aortic stenosis. Weidemann et al. showed that myocardial fibrosis is an important morphological substrate of unfavorable postoperative clinical outcome in patients with severe aortic stenosis, which is not reversible after aortic valve replacement over the 9 months of follow-up and could therefore be a parameter to be included in a pre-operative assessment of patients [30]. Importantly, in our study, LGE was present in 37% of cases, including 32.6% of ischemic type which may have further impact on the clinical outcome [31].

## Conclusions

In elderly patients submitted to TAVI, CMR is feasible and allows a global appraisal of aortic and ventricular morphology and function, representing a viable complement to transthoracic echocardiography. CMR is not applicable in all patients due to general contraindications such as claustrophobia and previous implantation of different devices. Therefore, its performance should be directed to patients in whom conventional TTE does not give a sufficient amount of information to drive decision making in TAVI.

## List of abbreviations

AVA: aortic valve area; CMR: cardiovascular magnetic resonance; LGE: late gadolinium enhancement; LVEF: left ventricular ejection fraction; LVOT: left ventricular outflow tract; MDCT: multidetector row computer tomography; SSFP: steady-state free precession; TAVI: transcatheter aortic valve implantation; TEE: trans-esophageal echocardiography; TTE: trans-thoracic echocardiography.

## Competing interests

The authors declare that they have no competing interests.

## Authors' contributions

ALM conceived and designed the study, participated in data analysis, interpretation and manuscript drafting and was responsible for the final manuscript draft. AS participated in study design, data analysis and interpretation, statistical analysis and manuscript drafting. DC participated in data analysis and interpretation, statistical analysis and manuscript drafting. AS, GP, WD, AM, GC, MF and RP performed additional data analysis. GPU, CP and CT participated in revising the manuscript critically for important intellectual content.

All authors read and approved the final manuscript.
